# Narrative review of the prevalence and distribution of acute pain in children in the self‐care setting

**DOI:** 10.1002/pne2.12085

**Published:** 2022-08-08

**Authors:** Nutan Shinde, Dipak J. Kanabar, Lisa J. Miles

**Affiliations:** ^1^ Reckitt Benckiser plc (Global Headquarters) Berkshire UK; ^2^ Evelina Children's Hospital London UK

**Keywords:** acute pain, Analgesia, prevalence, paediatric, pain assessment, self‐care

## Abstract

Acute pain among children is common, yet it may be underestimated and undertreated if the pain is not recognized. Assessing and managing pediatric pain can be complicated, and as such, measuring the prevalence of acute pain in children can be challenging. We sought to provide a consolidated review of the available data on the prevalence of commonly occurring acute pain in children in the self‐care setting. An extensive literature search was performed to determine the prevalence of acute pain at multiple bodily locations in children aged between 3 months and 18 years. We considered the influence of age, sex, and sociodemographic factors on prevalence estimates. We also sought to identify some of the challenges involved in assessing and managing pediatric pain, thus shedding light on areas where there may be clinical and medical unmet needs. In general, a high prevalence of acute pain in children was detected, particularly headache, menstruation‐related pain, and dental and back pain. Older age, female sex, and lower socioeconomic status were associated with increased pain prevalence. Risk factors were identified for all pain types and included psychological issues, stress, and unhealthy lifestyle habits. Owing to the heterogeneity in study populations, the prevalence estimates varied widely; there was also heterogeneity in the pain assessment tools utilized. The paucity of information regarding pain prevalence appears to be out of proportion with the burden of acute pain in children. This could indicate that clinicians may not be equipped with an optimal pain management strategy to guide their practice, especially regarding the use of developmentally appropriate pain assessment tools, without which prevalence data may not be captured. If acute pain is not accurately identified, it cannot be optimally treated. Further investigation is required to determine how the information from prevalence studies translates to the real‐world setting.

## 1 INTRODUCTION

Acute pain is described as lasting for 3 months or less,^1^ and is usually caused by illness, injury, or medical procedure.^2^ Acute pain is commonly experienced by children, although it may be underestimated and undertreated because of the inability of young children to understand and effectively communicate their symptoms, and the perception by adults and healthcare professionals that the pain is not serious enough to warrant intervention.^3^, ^4^, ^5^ Pain is a subjective phenomenon and unique to an individual. Assessing pediatric acute pain can be problematic, and there are numerous pain assessment tools whose use depends on the child's age, cognitive and communication skills, and pain location. There is currently no evidence to suggest that one tool is superior to the others.^6^ All relevant information pertaining to the child's situation must be considered,^1^ to allow the pain to be recognized and treated appropriately. The effect of untreated pain on children's daily activities and quality of life can be significant,^3^ and some may experience a continuation of their pain and its sequelae from childhood into adulthood.^1^

Measuring the prevalence of acute pain in a pediatric population can be challenging, and various factors relating to trial methodology can influence the quality and quantity of data, such as small sample sizes, recruitment of pediatric patients across a wide age range, and ethical challenges that researchers need to consider when carrying out pediatric clinical trials.^7^, ^8^ Studies may also involve subsets that are not applicable to the general population.^3^

The primary aim of this review was to provide a consolidated review of the available data on the prevalence of commonly occurring acute pain, stratified by pain location, in children aged between 3 months and 18 years being treated in the self‐care environment. We also sought to determine the effects of age, sex, and sociodemographic factors on pain prevalence, and elucidate the key challenges involved in assessing and managing acute pain in pediatric populations.

## 2 METHODS

### 2.1 Literature review

A literature search was performed on 12 August 2020 using PubMed® (including MEDLINE®), with a search string including the following terms: (pain [Title/abstract]) AND (baby [Title/abstract] OR infant [Title/abstract] OR child* [Title/abstract] OR adolescent [Title/abstract] OR teenager [Title/abstract] OR pediatric [Title/abstract] OR pediatric [Title/abstract]) NOT (pregnant OR pregnancy OR labour OR labor OR chronic pain OR cancer pain OR hospital OR intensive care OR palliative OR case report) AND (prevalence [Title/abstract] OR distribution [Title/abstract] OR epidemiolog* [Title/abstract]). Filters: English and Human. Dates were limited to 10 years. This search produced 615 hits.

Another literature search was performed on 28 August 2020 using EmBase. The search terms can be found in the Supplementary materials. This search produced 776 hits.

### 2.2 Selection criteria

We performed a literature search and review and have provided a narrative account of the prevalence of acute pain in children in the self‐care setting. After removing the duplicate records, we screened the titles and abstracts of the retrieved references against the predefined inclusion and exclusion criteria. The inclusion criteria were epidemiological studies of any design and duration which included experimental studies (randomized controlled trials, field trials, and community trials) and observational studies (descriptive and analytical studies, except for case reports), and meta‐analyses and systematic reviews that specifically reported data on self‐limiting, self‐treated acute pain (i.e., not from a chronic pain condition); full publications in English from the last 10 years (August 2010 to August 2020) including children 3 months to <18 years of age being treated in a self‐care environment for acute pain of 1‐week duration or less; treatments were over‐the‐counter (OTC) pain medications or no treatment in a self‐care environment; and the outcomes were prevalence, type, and severity of acute pain in a self‐care environment in children. The definition of self‐care from the World Health Organization was used: “the ability of individuals, families and communities to promote health, prevent disease, maintain health, and to cope with illness and disability with or without the support of a healthcare provider”.^9^ Children who suffered from acute pain irrespective of a chronic condition or disability were included. The exclusion criteria were articles for which full text was not available, were not in English, case reports, conference abstracts or posters, studies with a focus on hospitalized patients, patients with a chronic pain condition, prescription pain relief, cancer pain, pain related to parasites, or reports published in the gray literature. The search results were screened by a nonblinded reviewer to exclude articles that did not coincide with the inclusion criteria.

### 2.3 Critical assessment

Possible articles of interest were retrieved, reviewed and relevant data extracted into an Excel spreadsheet. Dual validation was not performed as the intention was not to provide a quantitative meta‐analysis. Each article was critically evaluated according to the following where applicable, with data for each category being evaluated within a separate column of the spreadsheet: study design; study endpoints; patient population; prevalence of acute pain; prevalence by age and sex; socioeconomic differences; pain scale/assessment tool used; self‐ or proxy‐reporting; causes of or risk factors for pain; management strategies, such as self‐medication (pharmacological or nonpharmacological) or healthcare professional intervention (self‐care could be with or without the support of a healthcare professional^9^); type and dosage of painkillers used; impact on quality of life, school performance and absenteeism; psychological factors and behavioral changes.

### 2.4 Assessment of pain

Although the questionnaires used to obtain information from the children varied between studies in terms of structure, content, and length, most questionnaires requested specific information on pain characteristics, such as type, location, timing, intensity, duration, and frequency, and required either yes/no or multiple‐choice responses. Details about specific criteria on which questionnaires were based can be found within each pain subtype. Most questionnaires were self‐reported; otherwise, parents/carers supported with the pain assessment.

## 3 RESULTS

**FIGURE 1:**
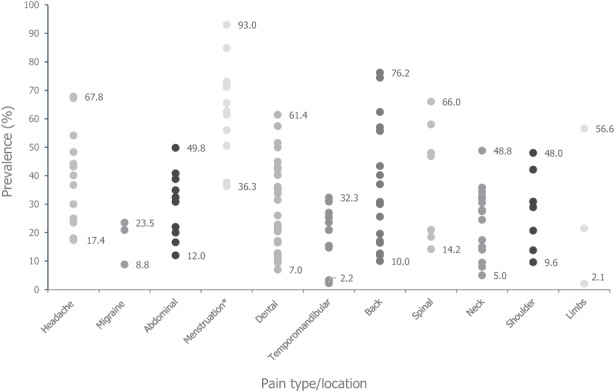
Dot plot showing prevalence estimates from individual studies for each pain type/location. *Female population only.

Of the 1391 articles that were screened, data related to acute pain prevalence were extracted from 131 studies for inclusion in this narrative review, consisting of worldwide data from studies of different types of acute pain in children, including headache, migraine, abdominal pain, functional gastrointestinal disorders, menstruation‐related pain, dental pain, temporomandibular pain, musculoskeletal pain in the back/neck/shoulders/spine, and musculoskeletal pain in the limbs (Figure 1).

### 3.1 Prevalence of headache

A clinical classification system for the diagnosis of headaches in children and adolescents was proposed by Rothner in 1993. Headaches are divided into five temporal patterns: acute, acute and recurrent, chronic and nonprogressive, chronic and progressive and “mixed” types.^10^ Migraine is the most common type of acute recurrent headache in children, with episodes of head pain separated by symptom‐free intervals.^11^, ^12^ Migraines are intense or severe headaches that are often accompanied by a variety of sensory, affective, cognitive, and autonomic symptoms, such as nausea, vomiting, periocular pain, photophobia, and phonophobia.^11^, ^13^ In this narrative review, we did not take into account chronic progressive and chronic nonprogressive headaches or any mixed type of headache pattern.

In this analysis, some questionnaires used to assess headache were based on diagnostic criteria for headaches, such as the International Classification of Headache Disorders (ICHD)/International Headache Society^14^, ^15^, ^16^, ^17^; the Health Behavior of School‐aged Children (HBSC) symptom checklist^18^, ^19^, ^20^, ^21^, ^22^, ^23^; or other validated or previously used tools.^24^, ^25^, ^26^, ^27^, ^28^, ^29^, ^30^

Prevalence estimates have previously indicated that headache is one of the most commonly reported types of pain in children.^31^ In 15 studies included in this analysis, estimates of headache prevalence ranged from 17.4% to 67.8%^3^, ^14^, ^19^, ^21^, ^22^, ^23^, ^24^, ^25^, ^26^, ^27^, ^28^, ^29^, ^30^, ^32^, ^33^ (Table 1). In five studies where an age breakdown was available, there were clear increases in head pain prevalence with increasing age,^3^, ^18^, ^23^, ^24^, ^32^ although in the report by Keeratisiroj et al.^28^, this age‐related increase occurred in girls but not boys from the ages of 9–14 years (48.8%) to 15–19 years (60.3%). Only one study by Du et al.^3^ evaluated headache in children aged under 5 years (Table 1). Headache was reported more frequently in girls than in boys in 13 studies where a split according to sex was available.^3^, ^18^, ^20^, ^22^, ^23^, ^24^, ^25^, ^26^, ^27^, ^28^, ^29^, ^30^, ^33^ Indeed, in female‐only studies, the prevalence rates were higher than in the mixed‐sex studies, ranging from 67.2% to 87.7%.^16^, ^17^ There was no difference in headache prevalence between different socioeconomic groups.^3^, ^24^

**TABLE 1:** Headache prevalence rates by individual study

Studies (patient number)	Country	Type of study	Patient number by age and sex	Overall prevalence	Prevalence by age and sex
Arruda et al. (2010) (*N* = 1906)	Brazil	Population‐based study	5–12 years old	Migraine: 3.76% Probable migraine: 17.1% Infrequent episodic tension‐type headache: 2.3% Frequent episodic tension‐type headache: 1.6% Probable tension‐type headache: 13.5%	Not reported
Kröner‐Herwig et al. (2010) (*N* = 2342)	Germany	Population‐based study	11–14 years old Girls: *N* = 1157 Boys: *N* = 1185	67.8%	11 and 12 years old: 65.7% 13 and 14 years old: 70.1% Girls: 73.2% Boys: 62.6%
Du et al. (2011) (*N* = 14 836)	Germany	Population‐based study	3–6 years old: *N* = 3875 7–10 years old: *N* = 4148 11–13 years old: *N* = 3076 14–17 years old: *N* = 3737 Girls: *N* = 7267 Boys: *N* = 7569	44.2%	3–6 years old: 20.2% 7–10 years old: 40.8% 11–13 years old: 43.7% 14–17 years old: 66.3% Girls: 48.5% Boys: 40.1%
Kröner‐Herwig et al. (2011) (*N* = 2219)	Germany	Longitudinal study	10–17 years old Girls: *N* = 1117 Boys: *N* = 1102	40.1%	Not reported
van Gessel et al. 2011 (*N* = 2025)	Germany	Population‐based longitudinal study	9–14 years old Girls: *N* = 1019 Boys: *N* = 1006	35.5–43.2%	Not reported
Romero‐Acosta et al. (2013) (*N* = 2558)	Spain	Cross‐sectional study	8–10 years old: *N* = 596 11–12 years old: *N* = 873 13–14 years old: *N* = 862 15–16 years old: *N* = 227 Girls: *N* = 1297 Boys: *N* = 1278	48.3%	Girls 8–10 years old: 13.3% 11–12 years old: 12.2% 13–14 years old: 38.5% 15–16 years old: 37.6% Boys 8–10 years old: 13.2% 11–12 years old: 6.3% 13–14 years old: 17.6% 15–16 years old: 19.8%
Sollerhed et al. (2013) (*N* = 206)	Sweden	Cross‐sectional study	*Numbers are calculated from percentages reported in the paper* 8 years old: *N* = 37 9 years old: *N* = 47 10 years old: *N* = 45 11 years old: *N* = 43 12 years old: *N* = 33 Girls: *N* = 92 Boys: *N* = 114	30%	Girls: 38% Boys: 23%
Lima et al. (2014) (*N* = 204)	Brazil	Cross‐sectional study	10–19 years old Girls: *N* = 204	87.7%	Not reported
Swain et al. (2014) (*N* = 404 206)	International	Cross‐sectional study	9.8–17.3 years old Girls: *N* = 207 112 Boys: *N* = 197 094	54.1%	11 years old: 48.3% 13 years old: 54.8% 15 years old: 59.4% Girls: 60.4% Boys: 47.5%
Gobina et al. (2015) (*N* = 36 762)	International	Cross‐sectional study	15 years old Girls: *N* = 18 499 Boys: *N* = 18 263	>30%	Not reported
Østerås et al. (2016) (*N* = 422)	Norway	Cross‐sectional study	15–16 years old Girls: *N* = 218 Boys: *N* = 204	18%	Girls: 24.8% Boys: 10.8%
Ramírez‐Vélez et al. (2017) (*N* = 7402)	Colombia	Cross‐sectional study	9–17.9 years old Girls: *N* = 4140 Boys: *N* = 3280	36.7%	Girls: 27.8% Boys: 47.7%
Grimby‐Ekman et al. (2018) (*N* = 11 374; 2008 cohort)	Sweden	Cross‐sectional study	Girls: *N* = 5758 Boys: *N* = 5616	25%	Girls: 35% Boys: 15%
Jurišić et al. (2018) (*N* = 5741)	Croatia	Cross‐sectional study	11 years old: *N* = 1792 13 years old: *N* = 2003 15 years old: *N* = 1946 Girls: *N* = 2857 Boys: *N* = 2884	23.5%	Girls 11 years old: 21.3% 13 years old: 36.0% 15 years old: 42.7% Boys 11 years old: 30.9%, 13 years old: 36.9% 15 years old: 32.3%
Keeratisiroj et al. (2018) (*N* = 2750)	Thailand	Cross‐sectional study	10–14 years old: *N* = 1676 15–19 years old: *N* = 1074 Girls: *N* = 1374 Boys: *N* = 1376	43.4%	Girls: 9–14 years old: 48.8% 15–19 years old: 60.3% Boys: 9–14 years old: 33.0% 15–19 years old: 33.2%
ALBashtawy et al. (2019) (*N* = 754)	Jordan	Cross‐sectional study	16–17 years old: *N* = 451 >17–18 years old: *N* = 303 Girls: *N* = 352 Boys: *N* = 402	Overall headache prevalence: 67.2% Tension‐type headache: 19.0% Migraine: 8.8% Unknown type of headache: 39.0%	Girls (tension type, migraine) 16–17 years old: 17.7%, 4.1% >17–18 years old: 36.4%, 7.5% Boys (tension type, migraine) 16–17 years old: 10.3%, 7.7% >17–18 years old: 18.1%, 19.9%
Gustafsson et al. (2019) (*N* = 568)	Finland	Population‐based longitudinal cohort study	10–15 years old	Overall (frequent, weekly) 10 years old: 7%, 13% 12 years old: 6%, 11% 15 years old: 8%, 20%	Girls (frequent, weekly) 10 years old: 9%, 17% 12 years old: 8%, 13% 15 years old: 11%, 24% Boys (frequent, weekly): 10 years old: 4%, 10% 12 years old: 5%, 8% 15 years old: 5%, 14%
Hena et al. (2019) (*N* = 878)	Sweden	Cross‐sectional study	13–15 years old Girls: *N* = 497 Boys: *N* = 293	Not reported	Girls, Boys: Without depressive symptoms: 33.3%, 18.1% With depressive symptoms: 63.7%, 43.6%

The prevalence of migraine ranged from 8.8 to 23.5% in four studies (episodic and probable migraine figures combined for Arruda et al)^14^, ^16^, ^17^, ^27^; migraine occurred more frequently during menstruation,^16^, ^17^ which is in keeping with the knowledge that in early childhood, migraine is more commonly seen in boys than girls, but from puberty onwards, the prevalence in females rises rapidly.^11^, ^12^, ^34^

Two studies found that psychological symptoms, such as depression and anxiety, were significantly associated with headache.^18^, ^24^ A positive family history of headache was reported by 53.2% of children in one study by ALBashtawy et al.^27^ With advancing age, children are increasingly exposed to risk factors and triggers, such as social pressures and educational demands, and certain unfavorable lifestyle habits, such as smoking and drinking alcohol, all of which may influence the frequency of headaches and compromise their quality of life.^19^, ^35^ The findings of our analysis suggest that headaches are more prevalent in adolescence than in early childhood and involve an interplay between biological, psychological, and socioenvironmental factors. A biopsychosocial approach to pain management would thus be of greater benefit than pharmacological treatment alone^36^, ^37^ and would allow the therapeutic strategy to be tailored to the factors that are unique to the individual.^36^ Behavioral treatment strategies can also help to ensure compliance with a pharmacological treatment, as well as supporting an improvement in longer‐term outcomes.^37^

In most cases, headaches do not indicate a serious underlying condition and can be managed at home with support from the parent/carer, and with advice from a pharmacist if needed.^38^ Reports of analgesic use in the studies in this analysis were limited. Du et al.^3^ noted that 10% of children used medications or went to see a doctor, although this was not specific to headache. ALBashtawy et al.^27^ reported that 26% of children sought help to relieve pain, and of those, 43.4% were advised to take analgesics. In the study by Lima et al.^17^, a need for pain medication was reported by 70.3% of adolescents (female‐only study), although only 26.2% sought medical attention, indicating that most subjects self‐medicated. Adebayo et al.^16^ noted that females with menstruation‐related headaches were significantly more likely to consult a doctor (53.8% vs 30.9%; *p* = 0.03) and were more likely to self‐medicate (76.9% vs 59.1%; not significant) compared with females with nonmenstruation‐related headaches. They also found that the most used medication was paracetamol (67.5% for all primary headaches). In a large international comparative study by Gobina et al.^22^, almost half of 15‐year‐old adolescents used medicine for headache. It is important to ensure that a child with a headache receives appropriate treatment, including the right medication at the correct dose, in a timely manner. However, overuse of medications is itself a contributing factor to headaches in children.^39^

It is evident that some of the risk factors for headache are preventable, and by adopting nonpharmacological as well as pharmacological therapies for selected patients and certain headache types, the frequency of headaches could be reduced. Although there was no evidence that participants were advised to make lifestyle changes in this analysis, any pragmatic strategy to prevent acute headache and avoid any possible progression to a more chronic state has the potential to improve the overall health of children and adolescents. In this analysis, most headaches were self‐treated and advice from a primary care physician was rarely sought. Despite the availability of effective treatment, clear guidance on acute headache diagnosis and management is currently lacking.

### 3.2 Prevalence of abdominal pain

The cause of abdominal pain in children may originate from several organs or systems, including the stomach, intestines, appendix, liver, and gall bladder. Acute abdominal pain in children is also confounded by the range of underlying conditions that may be triggering the pain. These may be categorized as nonsurgical conditions, such as gastroenteritis, or surgical conditions, such as appendicitis.^40^ Acute abdominal pain can also occur with sickle cell disease, urinary tract infection, and short‐term constipation.^40^

In this analysis, some assessments of abdominal pain were based on the HBSC symptom checklist,^18^, ^20^, ^21^, ^22^, ^23^ ICHD‐2 criteria,^41^ or other validated or previously used tools.^24^, ^25^, ^30^, ^42^

Our analysis indicates that the prevalence of acute abdominal pain ranged from 12.0 to 49.8% in the general pediatric population in 10 studies^3^, ^21^, ^22^, ^23^, ^24^, ^25^, ^30^, ^32^, ^33^, ^42^ (Table 2). Two studies reported prevalence values of 1.5% and 57.0%,^41^, ^43^ although in both studies patients attended pediatric clinics or tertiary care centers. As these patients may not be reflective of the general population, these values have not been included within the overall estimate of prevalence of acute abdominal pain. Results from four studies where an age breakdown was available were mixed (Table 2).^3^, ^18^, ^23^, ^24^ Swain et al.^23^ noted a clear increase in prevalence from ages 11 to 13 to 15 years. Du et al.^3^, the only study that included children aged under 4 years, reported that the prevalence of abdominal pain did not increase across ages 3–6, 7–10, and 11–13 years, but was higher in children aged 14–15 years. Romero‐Acosta et al.^24^ found that prevalence was lower in those aged 11–12 years than in those aged 8–10 years, but higher in children aged 13–14 and 15–16 years. Gustafsson et al.^18^ demonstrated similar rates in children aged 10, 12, and 15 years. The inconclusive effect of age on abdominal pain prevalence is perhaps not surprising given the different age brackets studied, as well as the differences in methodology used and populations studied, which do not allow an accurate comparison. Abdominal pain was reported more frequently in girls than in boys in seven studies where a split according to sex was available.^3^, ^18^, ^22^, ^23^, ^24^, ^25^, ^33^

**TABLE 2:** Abdominal pain prevalence rates by individual study

Studies (patient number)	Country	Type of study	Patient number by age and sex	Overall prevalence	Prevalence by age and sex
Du et al. (2011) (*N* = 14 836)	Germany	Population‐based study	3–6 years old: *N* = 3875 7–10 years old: *N* = 4148 11–13 years old: *N* = 3076 14–17 years old: *N* = 3737 Girls: *N* = 7267 Boys: *N* = 7569	40.8%	3–6 years old: 39.9% 7–10 years old: 39.8% 11–13 years old: 36.4% 14–17 years old: 45.3% Girls 46.2% Boys 35.6%
Kroner‐Herwig et al. (2011) (*N* = 2219)	Germany	Longitudinal study	10–17 years old Girls: *N* = 1117 Boys: *N* = 1102	30.8%	Not reported
van Gessel et al. (2011) (*N* = 2025)	Germany	Population‐based longitudinal study	9–14 years old Girls: *N* = 1019 Boys: *N* = 1006	28.0–32.4%	More prevalent in girls than boys
Dore et al. (2012) (*N* = 1741)	Italy	Cross‐sectional study	6–15 years old	12%	Not reported
Motamed et al. (2012) (*N* = 100)	Iran	Cross‐sectional study	4–17 years old	57%	Not reported
Romero‐Acosta et al. (2013) (*N* = 2558)	Spain	Cross‐sectional study	8–10 years old: *N* = 596 11–12 years old: *N* = 873 13–14 years old: *N* = 862 15–16 years old: *N* = 227 Girls: *N* = 1297 Boys: *N* = 1278	38.8%	Girls, Boys: 8–10 years old: 14.8%, 13.2% 11–12 years old: 11.1%, 6.1% 13–14 years old: 27.8%, 12.5% 15–16 years old: 25.7%, 13.5%
Sollerhed et al. (2013) (*N* = 206)	Sweden	Cross‐sectional study	*Numbers are calculated from percentages reported in the paper* 8 years old: *N* = 37 9 years old: *N* = 47 10 years old: *N* = 45 11 years old: *N* = 43 12 years old: *N* = 33 Girls: *N* = 92 Boys: *N* = 114	22%	No differences between boys and girls
Swain et al., (2014) (*N* = 404 206)	International	Cross‐sectional study	9.8–17.3 years old Girls: *N* = 207 112 Boys: *N* = 197 094	49.8%	11 years old: 45.1% 13 years old: 50.8% 15 years old: 53.4% Girls: 59.5% Boys: 39.4%
Gobina et al. (2015) (*N* = 36 762)	International	Cross‐sectional study	15 years old Boys: 18263 Girls: 18499	20%	More prevalent among girls than boys (except in Denmark and Greenland)
Ramirez‐Velez et al. (2017) (*N* = 7402)	Colombia	Cross‐sectional study	9–17 years old Girls: 4140 Boys: 3280	34.9%	Girls: 29.1% Boys: 42.0%
Grimby‐Ekman et al., (2018) (*N* = 11 374; 2008 cohort)	Sweden	Cross‐sectional study	Girls: *N* = 5758 Boys: *N* = 5616	16.6%	Girls: 23% Boys: 10%
Gustafsson et al. (2019) (*N* = 568)	Finland	Population‐based longitudinal cohort study	10–15 years old	Overall (frequent, weekly) 10 years old: 5%, 9% 12 years old: 4%, 7% 15 years old: 4%, 8%	Boys (frequent, weekly) 10 years old: 4%, 7% 12 years old: 3%, 5% 15 years old: 4%, 6% Girls (frequent, weekly) 10 years old: 6%, 10% 12 years old: 5%, 10% 15 years old: 4%, 9%
Hena et al. (2019) (*N* = 878)	Sweden	Cross‐sectional study	13–15 years Girls: 497 Boys: 293	Not reported	Girls, Boys: Without depressive symptoms: 17.5%, 10.2% With depressive symptoms: 57.7%, 38.5%

In the studies reviewed here, one study showed an inverse association between lower abdominal pain and socioeconomic status,^3^ while another study indicated that there was no impact of social status on the prevalence of abdominal pain.^24^ Two studies found that psychological symptoms, such as depression and anxiety, were significantly associated with abdominal pain.^18^, ^24^ Identifying a social or psychological risk factors for acute abdominal pain could help to guide the healthcare professional or parent toward an appropriate intervention.

Very few reports of analgesic use were found. Although Du et al.^3^ reported that 10% of children used medications or went to see a doctor, this was not specific to abdominal pain. A study by Hena et al.^20^ found that the prevalence rate of analgesic consumption to ease headache and/or stomach pain was 57% among girls and 29% among boys. In the analysis by Gobina et al.^22^, one‐third of 15‐year‐old adolescents used medication for stomach ache.

Acute abdominal pain is common in children. Pain in the abdomen could signify a more serious condition^40^ and thus should be closely monitored. In this analysis, however, there was no consistent approach for the assessment of acute abdominal pain as a variety of tools were used, and the underlying causes were not investigated.

### 3.3 Prevalence of menstruation‐related pain

Pain related to menstruation, or dysmenorrhea, is a significant and widespread problem in adolescent females. While primary dysmenorrhea has no underlying pathology, secondary dysmenorrhea involves painful menstruation with underlying pathology.^44^ The most common cause of secondary dysmenorrhea is endometriosis, a chronic condition that is frequently underdiagnosed^45^ and can be considerably detrimental to quality of life.^44^

In the studies included in our analysis, self‐administered questionnaires were primarily used to elicit information from students. Most of the questionnaires were not based on specific diagnostic criteria or previously validated instruments, although two studies mentioned the use of pretested or validated tools.^46^, ^47^ Pain scales (faces or numeric rating) were used in around half of the studies.^47^, ^48^, ^49^, ^50^, ^51^, ^52^, ^53^ Interviews were conducted in three studies.^47^, ^49^, ^51^

Dysmenorrhea prevalence estimates in 14 studies ranged from 36.3% to 93.0%,^3^, ^46^, ^47^, ^48^, ^49^, ^50^, ^51^, ^52^, ^53^, ^54^, ^55^, ^56^, ^57^ although in 12 studies at least half of females were affected^3^, ^47^, ^48^, ^49^, ^50^, ^51^, ^52^, ^53^, ^55^, ^56^, ^57^ (Table 3). Variability in menstrual patterns and dysfunctions, as well as the postmenarche age of the subjects, may explain the wide ranges observed. Only two studies provided a more detailed age breakdown of dysmenorrhea prevalence rates: Du et al.^3^ reported that menstruation pain was experienced by 23.6% of girls aged 11–13 years, with the rate increasing considerably to 67.4% of those aged 14–17 years; Rigon et al.^55^ observed that in girls aged ≤14, 15, 16, 17, and 18 years, severe abdominal pain rates were 53%, 53%, 54%, 58%, and 55%, respectively. Another study by Wong et al.^53^ commented that girls with dysmenorrhea were significantly older than those without. A significant familial association was noted by Rani et al (2016), who discovered that girls were more likely to experience dysmenorrhea if a member of their family had previously suffered with it.^49^

**TABLE 3:** Menstruation‐related pain prevalence rates by individual study

Studies (patient number)	Country	Type of study	Patient number by age and sex	Overall prevalence	Prevalence by age	Intervention
Eryilmaz et al. (2010) (*N* = 1951)	Turkey	Descriptive study	13–18 years old	68.1–72.2%	Not reported	Not reported
Du et al. (2011) (*N* = 7267)	Germany	Population‐based study	3–6 years old: *N* = 3875 7–10 years old: *N* = 4148 11–13 years old: *N* = 3076 14–17 years old: *N* = 3737 Girls: *N* = 7267 Boys: *N* = 7569	50.5%	11–13 years old: 23.6% 14–17 years old: 67.4%	Used pain medication or visited a doctor: 10% (not specific to menstruation‐related pain)
Bata et al. (2012) (*N* = 596)	Jordan	Cross‐sectional study	12–20 years old	37.6%	Not reported	Used pain medication: 48%
Rigon et al. (2012) (*N* = 4892)	Italy	Cross‐sectional population study	13–21 years old	Menstrual‐related abdominal pain: 56% Dysmenorrhea: 6.2%	Menstruation‐related abdominal pain; dysmenorrhea 14 years old: 53%; 2% 15 years old: 53%; 5% 16 years old: 54%; 5% 17 years old: 58%; 5% 18 years old: 55%; 7%	Used pain medication: 42%
Pitangui et al. (2013) (*N* = 218)	Brazil	Cross‐sectional study	12–17 years old	73%	Not reported	Used pain medication: 63.8% Sought medical care: 13.4%
George et al. (2014) (*N* = 233)	India	Descriptive survey	12–17 years old	62.7%	Not reported	Used pain medication: 10.2% Bedrest: 74.0% Heat: 12.3% Massage: 10.2%
Abdelmoty et al. (2015) (*N* = 412)	Egypt	Cross‐sectional survey	11–19 years old	93%	Not reported	Used medication: 88% (not specific to menstruation‐related pain) Consulted a doctor: 8.9% Heat: 40.5% Rest: 21.0% Herbal drinks: 17.0% Exercise: 12.6%
Rani et al. (2016) (*N* = 300)	India	Cross‐sectional study	11–18 years old	61.33%	Not reported	Visited a doctor: 11.33% Used pain medication: 17.33% Rest: 74.0% Hot drink: 66.3% Heat: 3.3%
Yücel et al. (2018) (*N* = 2000)	Turkey	Cross‐sectional study	9–18 years old	84.8%	Not reported	Used pain medication: 34% Paracetamol: 65% Nonmedical methods of pain relief: 35.2% Sought help: 9.3%
Wong (2018) (*N* = 653)	China	Cross‐sectional descriptive study	13–19 years old	65.5%	Prevalence was higher in older than younger girls	Used pain medication: 16.6% Chinese herbs/traditional remedies: 1.4%
Armor et al. (2019) (*N* = 21 573)	International	Meta‐analysis	School and university‐aged women	71.1%	School‐age girls: 72.5% University‐age girls: 74.9%	Not reported
Sivakami et al. (2019) (*N* = 2564)	India	Cross‐sectional survey	>12 years old	36.3%	Not reported	Used pain medication: 21%
Wang et al. (2019) (*N* = 5813)	China	Survey	≤12 years old (*N* = 247) 13–15 years old (*N* = 2998) ≥16 years old (*N* = 2568)	72.1%	Not reported	Not reported

The percentage of subjects who took medication to treat their menstrual pain ranged widely from 10.2% to 63.8%.^47^, ^48^, ^49^, ^51^, ^53^, ^54^, ^55^ In some studies, alternative means of pain relief were described (Table 3).^47^, ^48^, ^49^, ^50^, ^53^ Rani et al.^49^ noted that girls in rural areas (village, native place, or county away from a city or urban or developed area) were more likely to choose natural methods to relieve their pain, whereas girls in urban areas tended to use medications; this may highlight a lack of access to medication for dysmenorrhea or a lack of education regarding the availability and safe use of these medications in rural areas.

In this analysis, an impact of menstruation‐related pain on daily activities was observed in 33.0–87.9% of subjects with dysmenorrhea.^47^, ^50^, ^51^, ^53^, ^57^ Furthermore, one study by Eryilmaz et al.^52^ found that suffering with dysmenorrhea had a detrimental effect on the subjects' relationships, with 31.8% of girls reporting problems with their families, and 32.1% having problems with friends.

Menstruation‐related pain is highly prevalent among adolescent females. Overall, these findings highlight numerous disadvantages that girls and young women with dysmenorrhea face from a physical and social perspective.

### 3.4 Prevalence of dental pain

Dental pain is a common experience for children and can have a significant impact at a functional, social, and psychological level. The prevalence of dental pain in the general pediatric population ranged from 7% to 61.4% in 31 studies.^3^, ^58^, ^59^, ^60^, ^61^, ^62^, ^63^, ^64^, ^65^, ^66^, ^67^, ^68^, ^69^, ^70^, ^71^, ^72^, ^73^, ^74^, ^75^, ^76^, ^77^, ^78^, ^79^, ^80^, ^81^, ^82^, ^83^, ^84^, ^85^, ^86^, ^87^ Two studies reported prevalence estimates of 70.3% and 80%^88^, ^89^; however, the estimate of 70.3% reported by Felipak et al.^88^ included rates of discomfort and pain, while the prevalence rate of 80% described by Lamenha‐Lins et al.^89^ was based on experience of dental pain at any point during the child's lifetime. As these two values may therefore not be representative of the prevalence of acute dental pain within the general population, they were not included in the prevalence range reported here. The analysis included a broad variety of populations, and there was significant heterogeneity in pain measurement tools, which may account for the wide range in dental pain prevalence observed in this analysis. Where standardized questionnaires were used to assess pain, the Dental Discomfort Questionnaire and Child Dental Pain Questionnaire were commonly adapted for use.^61^, ^68^, ^78^, ^87^, ^88^, ^90^, ^91^, ^92^

Findings from studies where an age breakdown was included were mixed, but generally indicated that the prevalence of dental pain increased with age.^3^, ^64^, ^74^, ^76^, ^77^, ^81^ A wide range of different age brackets were used, which makes it difficult to draw comparisons. Only two studies evaluated the breakdown in prevalence of dental pain in children under 5 years old. Ortiz et al.^58^ reported that the prevalence increased across the ages of ≤1 year, 2–3 years, and 4–5 years (4.35%, 8.13%, and 16.86%, respectively), while Lemes et al (2015) saw no significant differences between children aged 2, 3, and 4 years old (9.4%, 9.8%, and 10.0%, respectively).^60^ Very few studies compared the prevalence of dental pain between younger and older children. Du et al (2011) observed that dental pain was experienced by 7.3% of 3–6‐year‐olds, rising to 14.3% of those aged 14–17 years.^3^ Similarly, Ghorbani et al.^64^ noted a prevalence of 17.4% in 4–8‐year‐olds, which rose to 27.3% in 13–17‐year‐olds. In contrast, Escoffié‐Ramirez et al.^81^ described similar prevalence rates in children aged 6–7, 8–10, and 11–12 years (48.7%, 51.4%, and 48.7%, respectively). Schuch et al.^76^ reported prevalence rates of 33.7%, 34.6%, 31.6%, 36.9%, and 45.4% in children aged 8, 9, 10, 11, and 12 years, respectively. Similar results were reported by Adeniyi et al.^77^, where the odds of reporting dental pain in children aged 8–12 years increased by 25.4% for every year increase in age. In contrast, Santos et al (2019) found no difference in prevalence rates between children aged 8, 9, and 10 years (51.6%, 52.8%, and 50.0%, respectively).^74^ The prevalence of dental pain appears to increase as children enter their teenage years; Freire et al (2019) reported that dental pain increased across the ages of 11–13, 14–16, and 17+ years (18.8%, 22.4%, and 29.7%, respectively).^67^

Dental pain was reported more frequently in girls in most studies where a breakdown according to sex was available^3^, ^58^, ^60^, ^64^, ^67^, ^70^, ^74^, ^76^; however, heterogeneity between studies and pediatric populations studied make it difficult to conclude whether female sex is a risk factor for oral pain.^68^, ^70^, ^71^

Several clinical, behavioral, and socioeconomic factors have been associated with dental pain. Multiple studies have reported a significant association between dental pain and caries,^58^, ^59^, ^61^, ^68^, ^69^, ^70^, ^71^, ^74^, ^75^, ^76^, ^77^, ^78^, ^88^, ^92^, ^93^ while molar incisor hypomineralization, ulceration, bleeding gums, missing teeth, filled teeth, and erupting molars have also been associated with dental pain.^68^, ^73^, ^74^, ^75^, ^88^ An unhealthy diet, including the consumption of sugary or soft drinks, fried foods, sweets, and alcohol, was also indicated as a risk factor for dental pain.^60^, ^66^, ^81^, ^90^ Socioeconomic factors, including low monthly income, lower parental education level, non‐nuclear family structure, living in crowded housing, and attending a public school, were also identified as risk factors.^3^, ^60^, ^64^, ^66^, ^67^, ^68^, ^70^, ^71^, ^75^, ^76^, ^77^, ^79^, ^81^, ^88^, ^90^, ^92^ Du et al (2011) noted that the association between social status and tooth pain could arise from insufficient hygiene within families of low socioeconomic status.^3^ Indeed, a link between dental hygiene and dental pain was identified in several studies. Brushing frequency, age that the child started brushing their teeth, age of first dental visit, whether the child had visited a dentist in the past year and oral health of the mother were all predictors of dental pain.^64^, ^70^, ^81^, ^94^ Dental anxiety is common in children and can lead to the avoidance of dental treatment and care, resulting in poor oral health and increasing the possibility of experiencing pain.^72^, ^84^, ^93^, ^95^ Dental pain, particularly if experienced during a previous dental appointment, and parental fear are associated with dental anxiety.^72^, ^93^, ^96^

Reports on medication and treatment were limited. Kalantary et al (2013) noted that toothache provoked a visit to the dentist in 40% of cases.^90^ In a cross‐sectional survey by Adeniyi et al.^77^, 31.9% of parents used medications at home for early management of dental pain and a few subjects reported using home therapies, such as warm saline baths; 21.7% reported visiting the dentist, 12.2% visited the doctor, 6.7% visited a nurse; and 13.0% sought advice from a pharmacist.

Early diagnosis and immediate treatment of oral conditions are essential, and regular dental checks can prevent conditions from worsening.^79^ Indeed, dental pain can significantly decrease quality of life and lead to distress for both the child and their family.^58^, ^62^, ^68^, ^87^, ^89^, ^90^, ^97^ Children may find that their ability to carry out daily activities such as eating, speaking, socializing, and sleeping is impaired if they are suffering with dental‐related pain,^59^, ^63^, ^65^, ^68^, ^74^, ^87^, ^90^, ^98^, ^99^ with daily activities being impacted in 21.5–73.3% of those who reported pain.^59^, ^65^, ^68^, ^77^, ^90^, ^99^ The consequences of untreated dental pain may follow a child into adulthood, and even have a detrimental effect on future employment prospects.^74^

Despite being an often preventable and treatable problem, dental pain represents a major public health issue.^58^, ^65^ Considering that children of low socioeconomic status are at increased risk of experiencing dental problems, further action to reduce social inequalities could improve oral health in this demographic.^65^, ^71^, ^79^ Specific educational interventions aimed at improving the awareness and prevention of oral disease in children, families, and healthcare services are also required.^58^, ^65^

### 3.5 Prevalence of temporomandibular pain

Temporomandibular disorders (TMDs) are disorders of the temporomandibular joint, the masticatory muscles and their associated structures. TMDs are characterized by pain in the jaw and surrounding muscles, joint noises, and restricted mandibular movement.^100^

This analysis found that clinical examinations were regularly used in the assessment and diagnosis of temporomandibular (TM) pain.^101^, ^102^, ^103^, ^104^, ^105^ Some questionnaires were based on validated^101^, ^102^, ^103^, ^106^, ^107^, ^108^ or previously used^109^ tools.

The prevalence of TM pain in this analysis ranged from 2.2% to 32.3% in 11 studies.^101^, ^102^, ^103^, ^104^, ^105^, ^106^, ^107^, ^109^, ^110^, ^111^, ^112^ Four studies provided more detail on the influence of age on TM pain prevalence rates, although results were mixed.^103^, ^107^, ^109^, ^111^ In studies by De Paiva Bertoli et al (2018) and Hirsch et al (2012), there was no significant change in TM pain with increasing age.^103^, ^107^ However, Marpaung et al (2018) reported that the prevalence rates for pain‐related TMDs in subjects aged 7–12 and 13–18 years were 23.4% and 36.9%, respectively.^109^ While TM pain experienced while chewing was similar across age groups in the study by Inglehart et al.^111^, pain when opening the mouth wide was experienced by 21.9%, 20.7%, and 13.6% of subjects aged 5–7, 7–9, and 9–11 years, respectively. Thus, there is a lack of conclusive data concerning how prevalence of TM pain changes with age. No studies investigated the presence of TM pain in children aged under 5 years. In four studies where a split in prevalence estimates according to sex was available, TM pain was reported more frequently in girls than in boys^103^, ^105^, ^107^, ^111^; however, three studies claimed that there was no difference in the prevalence between the sexes.^102^, ^109^, ^112^ Therefore, it cannot be concluded with certainty that female sex is a risk factor for the occurrence of TM pain. There was no difference in TM pain prevalence between different socioeconomic groups.^109^, ^111^, ^112^

Some studies in this analysis did not report or find any causative factors relating to the presence of TM pain or TMD symptoms^101^, ^105^, ^108^, ^110^, ^111^; however, in other studies, associations between TM pain and bruxism,^102^, ^109^ parafunctional habits,^102^, ^106^, ^109^ other bodily pain,^102^, ^112^ and pubertal growth^107^ were noted. Bite disorders were associated with TM pain in two studies.^104^, ^106^ Psychological factors were deemed to be risk indicators for TM pain in the study by Marpaung et al.^109^. Inglehart et al.^111^ also found that the presence of TMD symptoms had a negative impact on children's psychological and social well‐being.

Reports of medical interventions were scarce—in one study by Vierola et al.^112^, 61% of subjects used pain medication and 16% visited a physician, but this was not specific to those with TM pain; Hirsch et al.^107^ found that 14.4% of subjects used pain medication more than once per week, but this included subjects with pain outside the face and those experiencing TM pain.

The range in the prevalence of TM pain is perhaps not surprising given the variety of methods and diagnostic criteria used in the different studies. Given the lack of conclusive evidence on causative factors and information regarding medical interventions, further research into this pain type is clearly warranted as strategies do not appear to be in place for its effective management. Early intervention when symptoms and signs of TMD appear during childhood is fundamental as TM pain may continue into adulthood.^103^

### 3.6 Prevalence of musculoskeletal pain in the back, neck, shoulders, and spine

Pain related to the back, neck, shoulders, and spine is a significant problem in children, especially in young adolescents of secondary school age. In terms of pain assessment, many studies in this analysis asked children if they had experienced pain in regions clearly indicated on a body map or mannequin,^28^, ^113^, ^114^, ^115^, ^116^, ^117^, ^118^, ^119^, ^120^, ^121^, ^122^ or instructed them to indicate where the pain was located using pain drawings.^123^ Some questionnaires were based on diagnostic criteria such as the HBSC symptom checklist,^21^, ^22^ or other previously validated tools and questionnaires.^28^, ^115^, ^122^, ^124^, ^125^, ^126^, ^127^

In this analysis, the prevalence of back pain was 10.0–76.2% in 17 studies^3^, ^21^, ^22^, ^23^, ^25^, ^29^, ^30^, ^114^, ^119^, ^126^, ^128^, ^129^, ^130^, ^131^, ^132^, ^133^, ^134^ (Table 4), neck pain was 5.0–49.0% in 15 studies,^28^, ^29^, ^113^, ^114^, ^119^, ^122^, ^125^, ^127^, ^135^, ^136^, ^137^, ^138^, ^139^, ^140^, ^141^ shoulder pain was 9.6–48.0% in eight studies,^28^, ^29^, ^113^, ^114^, ^122^, ^138^, ^141^, ^142^ and spinal pain was 14.2–66.0% in six studies.^116^, ^118^, ^139^, ^143^, ^144^, ^145^ In studies where a breakdown of pain in different regions of the back was available, the prevalence of pain ranged from 4.0–57.0% for the lower back in 23 studies,^28^, ^114^, ^115^, ^117^, ^119^, ^120^, ^121^, ^122^, ^123^, ^124^, ^125^, ^127^, ^130^, ^135^, ^136^, ^137^, ^138^, ^141^, ^146^, ^147^, ^148^, ^149^, ^150^ 3.4–41.3% for the upper back in nine studies,^28^, ^114^, ^122^, ^123^, ^127^, ^130^, ^137^, ^138^, ^141^ and 4–35% for the middle back in four studies.^125^, ^130^, ^135^, ^151^ There was significant heterogeneity between studies in terms of the populations and age brackets studied, and differences in tools used to measure pain, which is likely to account for the wide ranges in prevalence observed in this analysis.

**TABLE 4:** Back pain prevalence rates by individual study

Studies (patient number)	Country	Type of study	Patient number by age and sex	Overall prevalence	Prevalence by age and sex	Risk/causative factors
Ayanniyi et al. (2011) (*N* = 3185)	Nigeria	Cross‐sectional study	10–13 years old: *N* = 510 14–16 years old: *N* = 2038 17–19 years old: *N* = 637 Girls: *N* = 1730 Boys: *N* = 1455	Point: 17% >3 months: 21%	10–13 years old: 18% 14–16 years old: 16% 17–19 years old: 18%	Prolonged sitting posture Frequent bending
Du et al. (2011) (*N* = 14 836)	Germany	Population‐based study	3–6 years old: *N* = 3875 7–10 years old: *N* = 4148 11–13 years old: *N* = 3076 14–17 years old: *N* = 3737 Girls: *N* = 7267 Boys: *N* = 7569	19.6%	3–6 years old: 2.6% 7–10 years old: 6.9% 11–13 years old: 18.4% 14–17 years old: 44.3% Girls: 22.6% Boys: 16.7%	Not reported
Kjaer et al., 2011 (*N* = 771)	Denmark	Longitudinal cohort study	9 years old: *N* = 479 13 years old: *N* = 439 15 years old: *N* = 443	Not reported	9 years old: 33% 13 years old: 28% 15 years old: 48%	Previously reported pain
Turk et al. (2011) (*N* = 190)	Slovenia	Cross‐sectional study	11–15 years old: *N* = 100 17–18 years old: *N* = 90	Not reported	11–15 years old: 43% 17–18 years old: 44%	Carrying school bag School chairs Intensive sport activity
van Gessel et al. (2011) (*N* = 2025)	Germany	Population‐based longitudinal study	9–14 years old Girls: *N* = 1019 Boys: *N* = 1006	20.6–30.8%	Girls 9 years: 21.5% 10 years: 15.0% 11 years: 19.6% Higher prevalence in girls than boys (values for boys not reported)	Not reported
Kędra et al. (2013) (*N* = 1089)	Poland	Cross‐sectional study	10–13 years old: *N* = 243 14–16 years old: *N* = 197 17–19 years old: *N* = 649 Girls: *N* = 547 Boys: *N* = 542	76.2%	10–13 years old: 64.2% 14–16 years old: 72.6% 17–19 years old: 81.8% Girls: 52.2% Boys: 47.8%	Physical work Sedentary position (>6 hours/day)
Sollerhed et al. (2013) (*N* = 206)	Sweden	Cross‐sectional study	*Numbers are calculated from percentages reported in the paper* 8 years old: *N* = 37 9 years old: *N* = 47 10 years old: *N* = 45 11 years old: *N* = 43 12 years old: *N* = 33 Girls: *N* = 92 Boys: *N* = 114	10%	No difference between girls and boys	Not feeling comfortable in school
Trigueiro et al. (2013) (*N* = 637)	Portugal	Cross‐sectional study	7 years old: *N* = 63 8 years old: *N* = 175 9 years old: *N* = 230 10 years old: *N* = 169 Girls: *N* = 323 Boys: *N* = 314	12.7%	7 years old: 15.9% 8 years old: 10.3% 9 years old: 11.7% 10 years old: 15.2%	School absences Parental pain Sleeping difficulties Inappropriate school furniture Postural deviations at the sagittal and frontal planes
Swain et al. (2014) (*N* = 404 206)	International	Cross‐sectional study	9.8–17.3 years old Girls: *N* = 207 112 Boys: *N* = 197 094	37%	11 years old: 27.4% 13 years old: 37.0% 15 years old: 46.7% Boys: 35.0% Girls: 38.9%	Not reported
Gobina et al. (2015) (*N* = 36 762)	International	Cross‐sectional study	15 years old Girls: *N* = 18 499 Boys: *N* = 18 263	<30%	In most countries, the prevalence of recurrent backache was higher among girls than boys	Female sex
Aprile et al., 2016 (*N* = 5318)	Italy	Cross‐sectional study	6–10 years old: *N* = 2459 11–14 years old: *N* = 1389 15–19 years old: *N* = 1470 Girls: *N* = 2599 Boys: *N* = 2719	62.4%	6–10 years old: 50.7% 11–14 years old: 67.7% 15–19 years old: 77.1%	Schoolbag carrying time Schoolbag load Female sex Adolescent age Recruitment area
Kamper et al. (2016) (*N* = 31 690– 125 483)	International	Qualitative data synthesis review	<18 years old	Point: 12% Lifetime: 40%	Girls are at higher risk of reporting back pain than boys, but some inconsistency between primary studies is noted	Psychological distress and psychosocial factors Female sex Smoking
Noll et al. (2016) (*N* = 1439)	Brazil	Cross‐sectional study	11–12 years old: *N* = 513 13–14 years old: *N* = 682 15–16 years old: *N* = 244 Girls: *N* = 674 Boys: *N* = 765	55.7%	11–12 years old: 53.2% 13–14 years old: 57.2% 15–16 years old: 57.0% Girls: 63.9% Boys: 48.5%	Female sex Parents with back pain Weekly frequency of physical activity Daily time spent watching television Studying in bed Sitting posture to write and use the computer Way of carrying the backpack
Østerås et al. (2016) (*N* = 422)	Norway	Cross‐sectional study	15–16 years old Girls: *N* = 218 Boys: *N* = 204	16.4%	Girls: 17.0% Boys: 15.7%	BMI (females only)
Ramírez‐Vélez et al., 2017 (*N* = 7402)	Colombia	Cross‐sectional study	9–17.9 years old Girls: *N* = 4140 Boys: *N* = 3280	25.6%	Girls: 23.3% Boys: 28.3%	Unhealthy cardiorespiratory fitness in girls Female sex
Gonçalves et al., 2018 (*N* = 350)	Brazil	Cross‐sectional study	Girls: *N* = 180 Boys: *N* = 170	40.2%	Girls: 55.0% Boys: 40.0%	Not reported
Grannemann et al. (2018) (*N* = 77)	Germany	Prospective cohort study	Control: *N* = 49 Girls: *N* = 24 Boys: *N* = 25 Backpacks reduced by 2 kg: *N* = 28 Girls: *N* = 16 Boys: *N* = 12	57%	Not reported	High backpack load
Kędra et al. (2019) (*N* = 11 424)	Poland	Cross‐sectional study	10–13 years old: *N* = 2944 14–16 years old: *N* = 4040 17–19 years old: *N* = 4440 Girls: *N* = 6252 Boys: *N* = 5172	74.4%	10–13 years old: 61.8% 14–16 years old: 74.0% 17–19 years old: 83.1% Girls: 82.8% Boys: 64.3%	More than 5 hours per day in a sedentary position Heavy backpack
Khalil et al. (2019) (*N* = 4667)	Iraq	Cross‐sectional study	9–10 years old: *N* = 2339 11–12 years old: *N* = 2152 13–14 years old: *N* = 176	43.4%	9–10 years old: 37% 11–12 years old: 48.1% 13–14 years old: 69.9% Girls: 46% Boys: 41%	Parent history of back pain Sleeping, sitting, and lifting postures Physical activity Sedentary activities Studying in bed

Where a breakdown was available for age, most studies suggested that back, neck, shoulder, and spinal pain increased with age. Few studies investigated the prevalence of pain in younger children. Du et al (2011) reported that the prevalence of back pain increased across the ages of 3–6, 7–10, 11–13, and 14–17 years (2.6%, 6.9%, 18.4%, and 44.3%, respectively),^3^ with similar results described by Wirth et al (2013) and Aprile et al (2016).^128^, ^143^ The overall age range across all other studies was between 9 and 19 years, with the majority reporting that the prevalence of pain generally increased with age for overall back,^23^, ^114^, ^131^, ^134^, ^135^ lower back,^28^, ^115^, ^117^, ^120^, ^121^, ^135^, ^146^, ^147^, ^148^ upper back,^28^ middle back (in girls only),^135^ neck,^28^, ^116^, ^135^, ^136^ and shoulder pain.^28^ However, Dianat et al.^122^ found that concurrent shoulder and lower back pain was significantly lower in older children (≥14 years) than in younger children (≤13 years), while Gheysvandi et al (2019) also observed no significant difference in neck and shoulder pain between children aged <10 years and ≥10 years.^113^ Musculoskeletal pain in the back, neck, shoulder, and spine was generally more common in girls in most studies where a split according to sex was available.^3^, ^22^, ^25^, ^113^, ^114^, ^116^, ^117^, ^119^, ^124^, ^125^, ^126^, ^128^, ^131^, ^132^, ^134^, ^135^, ^140^, ^143^, ^146^, ^148^

Back, neck, and spinal pain can decrease the quality of life for children or impact daily activities.^117^, ^118^, ^119^, ^125^, ^126^, ^130^, ^139^ Several physical and behavioral risk factors were identified for back, neck, and spinal pain in our analysis, including school bag use (carrying time, load, way of carrying),^28^, ^115^, ^128^, ^131^, ^133^, ^138^, ^143^, ^146^, ^152^, ^153^ posture,^129^, ^137^, ^146^ prolonged time spent in a sitting or sedentary position,^117^, ^130^, ^131^, ^132^, ^138^, ^139^, ^153^ school furniture,^152^ and activities that require bending.^113^, ^129^, ^130^, ^147^ Five studies indicated that computer and TV use were associated with increased risk of pain.^28^, ^114^, ^117^, ^153^, ^154^ The impact of exercise on back, neck, shoulder, and spine pain is uncertain—while a number of investigations indicated that lack of physical activity was a risk factor for pain,^30^, ^114^, ^136^ being physically active in sports activities was also associated with pain in this analysis.^114^, ^136^, ^152^ Khalil et al.^114^ found a significant association between exercise frequency and back pain, reporting that 51.1%, 4.8%, and 53.0% of children who exercised 1–2, 3–4, and 5+ days per week experienced back pain, respectively. Such findings highlight the importance of moderate exercise for the prevention of musculoskeletal pain. Conclusions on the impact of body weight or body mass index (BMI) on musculoskeletal pain could not be made due to conflicting results between studies.^128^, ^134^, ^145^

Few studies investigated the impact of socioeconomic status on back, neck, and shoulder pain, and findings were inconclusive. Two studies indicated that there was no association between social status and pain prevalence.^3^, ^136^ However, Joergensen et al.^125^ observed that children living in less‐educated or lower‐income families were more likely to experience spinal pain and, similarly, Stallknecht et al.^116^ found an association between parental education status and back and spinal pain. Psychological factors such as depressed mood, stress, distress, and poor general well‐being have also been associated with pain in the back, spine, shoulders, and neck.^116^, ^134^, ^140^ A familial association with back pain was noted in three studies.^118^, ^126^, ^146^ These findings therefore suggest that genetic, environmental, psychological, social, and physical factors may be associated with musculoskeletal pain.^116^, ^126^, ^134^, ^140^, ^143^, ^146^

Estimates for the prevalence of patients who sought medical care for their musculoskeletal pain ranged between 1.6% and 34%.^115^, ^116^, ^117^, ^123^, ^125^, ^130^, ^131^, ^132^, ^134^, ^135^, ^137^, ^139^, ^146^, ^148^ Of these patients, 0.8–20.3% visited a doctor,^115^, ^117^, ^137^, ^146^ 0.9–10.3% visited a nurse,^115^, ^146^ 3.5–44.4% saw a physiotherapist,^115^, ^131^, ^132^, ^146^ and 4.4–4.5% used medicines prescribed by a doctor.^131^, ^132^ A number of studies reported on the prevalence of self‐care measures for pain, such as rest (5.7–71.3%), OTC painkillers (12.3–27.0%), changing posture (19%), or interrupting training and activities (3.1%).^130^, ^131^, ^132^, ^146^ Given that musculoskeletal pain in the upper body can persist into adulthood,^33^, ^114^, ^116^ early treatment of pain is important to relieve discomfort for the child and minimize the consequences of pain during later life.^33^, ^116^, ^140^

It is clear that some musculoskeletal pain is preventable and that appropriate education on physical and behavioral risk factors is necessary to reduce back, neck, shoulder, and spinal pain.^126^, ^131^, ^141^, ^152^ However, musculoskeletal pain is a multifactorial condition that may benefit from a biopsychosocial approach to pain management.^36^, ^116^, ^118^, ^125^, ^126^, ^129^, ^143^, ^146^ Given the impact of musculoskeletal pain on childhood quality of life and daily functioning,^117^, ^118^, ^119^, ^125^, ^126^, ^130^, ^139^ longitudinal cohort studies are required to identify prognostic factors for pain and to investigate the effectiveness of pain interventions to provide evidence‐based treatments for children with back, spine, shoulder, and neck pain.^117^, ^134^, ^136^, ^141^, ^146^

### 3.7 Prevalence of musculoskeletal pain in the limbs

Limb pain is one of the most common types of pain experienced by children.^155^ It can be caused by specific and diagnosed conditions, including cerebral palsy, muscular dystrophy, arthritis, rheumatism, and diagnosed postural defects,^144^, ^155^, ^156^ but also by nonspecific causes such as playing sports/exercise.^28^, ^155^ Growing pains are a common cause for any type of limb pain in children.^155^ Although musculoskeletal limb pain may result from various causes including chronic conditions, this analysis only included estimates of prevalence from studies wherein the musculoskeletal pain was acute in nature.

Some studies in this analysis adopted previously validated questionnaires to assess musculoskeletal limb pain, such as the Standardized Nordic Questionnaire or Standardized Nordic Questionnaire for Osteomuscular Symptoms.^28^, ^141^, ^157^

The overall prevalence of musculoskeletal limb pain ranged from 2.1% to 56.6% in three studies,^155^, ^156^, ^158^ with overall estimates of 5.2–20% in the upper limbs and 8.2–33.3% in the lower limbs across four studies.^29^, ^145^, ^150^, ^159^ Within the upper limbs, the prevalence of musculoskeletal pain was 5.2–6.5% in the arms,^3^, ^29^ 16% in the hands,^144^ 4.2–9.0% in the elbows,^28^, ^141^ and 15.2–29.9% in the wrists.^28^, ^141^ In the lower limbs, the prevalence of pain was 18.2–31.9% in the ankles/feet in two studies,^28^, ^141^ 21.9% in the legs in one study,^3^ 16.3–64% in the knees in three studies,^28^, ^144^, ^157^ 15.3–30.4% in the thighs/hips in two studies,^28^, ^141^ and 3% in the heels in one study.^156^ Joint pain was reported in 4.1–12.3% of children in one study.^160^

The prevalence of musculoskeletal limb pain appeared to increase with age, according to two studies. Du et al.^3^, which was the only study to investigate limb pain in children aged under 5 years, observed that leg pain increased from 16.3% in 3–6‐year‐olds to 26.3% in 14–17‐year‐olds, while arm pain increased from 2.1% to 10.4%. Similarly, Saes et al.^157^ reported that knee pain increased from 14.5% in 10–11‐year‐olds to 34.7% in 15–17‐year‐olds. However, conflicting evidence was published in one study by Keeratisiroj et al.^28^, who observed that the prevalence of knee, ankle, and foot pain was higher in children aged 10–14 years old compared with those aged 15–19 years. Differences between studies in the populations and age brackets used are likely to account for the discrepancies reported in this analysis.

Four studies reported that pain prevalence was similar across sexes with respect to lower and upper body pain^3^, ^29^, ^157^, ^158^; however, three investigations indicated that the prevalence of limb pain was slightly higher in boys than girls,^155^, ^159^ particularly in the knees, ankles, and feet.^28^ Therefore, it is difficult to conclude whether males are more likely than females to experience musculoskeletal pain in the limbs.

Musculoskeletal pain in the limbs can interfere with daily activities such as studying, sleeping, and playing sports, and can lead to functional impairment.^144^, ^145^, ^157^ Two studies identified being overweight as a risk factor for musculoskeletal pain in the upper limbs, knees joints, and feet^144^, ^145^; however, firm conclusions regarding the impact of body weight could not be made due to conflicting evidence presented by Saes et al.^157^, who did not find an association between the presence of knee pain and increased body weight. Though lower limb pain has traditionally been associated with traumatic pain rather than stress‐associated pain, Østerås et al (2015) described how perceived stress was similar in children with lower extremity pain and neck/shoulder pain, suggesting that stress mechanisms can also influence pain that would typically be associated with activity.^29^, ^161^ Such findings demonstrate the necessity of taking psychological factors into account when managing pain in the lower extremities.

Two studies indicated that there was an association between social status and limb pain prevalence. Golding et al.^158^ observed that growing pains in 8‐year‐olds were more likely to be reported by children with mothers with lower educational achievements, from a lower social class and lower parity; smoking during pregnancy and exposure to environmental smoke during childhood were also identified as risk factors. Similarly, Bishop et al.^155^ noted that limb and growing pains were less likely to be reported in mothers who had a higher education status. Further studies are required to fully determine this relationship.

Few studies discussed the treatment of musculoskeletal limb pain, and those that did discussed musculoskeletal pain overall, including back and spinal pain. Silva et al.^145^ reported that 32.1% of subjects sporadically used and 11.1% frequently used analgesics for musculoskeletal pain, which included upper limb pain. Słowińska et al.^144^ noted that 50%, 41%, and 5% of children consulted a doctor/pediatrician, orthopedician, or rheumatologist, respectively, for musculoskeletal pain (knee, hand, and spine pain).

Musculoskeletal pain in the limbs is highly prevalent in children and adolescents, and is associated with biological, physical, psychological, and socioeconomic factors.^28^, ^144^, ^145^, ^155^, ^156^, ^158^ However, literature on musculoskeletal pain in children is scarce, highlighting the need for further studies on limb pain and its associated factors to facilitate preventative measures, early diagnosis, and effective interventions for treatment.

## 4 DISCUSSION

This review aimed to provide a consolidated summary of the available data on the prevalence of commonly occurring acute pediatric pain in the self‐care environment, stratified by pain location.

We also sought to determine the influence of age, sex, and sociodemographic factors on pain prevalence, and to highlight some of the key challenges involved in assessing and managing acute pediatric pain.

The results of this review indicate that there is a high prevalence of acute pain in children, particularly headache, dental and back pain (Figure 1). Menstruation‐related pain is also a very common problem in females following menarche. Owing to the heterogeneity in study populations, age ranges, and methodology, the prevalence estimates vary widely. Prevalence studies generally only involve small subsets that are not applicable to the general population; therefore, there is likely an overall level of bias in the prevalence estimates reported here. There were clear increases in prevalence of some acute pain conditions with increasing age, notably headache, dental, back, neck, shoulder, spinal and limb pain. Only three studies reported pain prevalence data in preschool children.^3^, ^58^, ^60^ Pain frequency was found to be higher in females than in males for headache, abdominal, dental, back, neck, shoulder, and spinal pain; the only pain type that appeared to occur more frequently in males than females was musculoskeletal limb pain. Most studies only considered one socioeconomic group within one geography; a higher pain prevalence in those with a lower socioeconomic status was observed with dental and musculoskeletal pain.

Pain risk factors and triggers responsible for increasing the frequency of pain were identified for all pain types, including psychological issues such as depression and anxiety for headache and abdominal pain, family history of dysmenorrhea, low socioeconomic status and poor diet for dental pain, bruxism and parafunctional habits for TM pain, and stress and poor general well‐being for musculoskeletal pain. However, not all studies sought to elucidate any risk factors or potential biological cause associated with the pain. Evidence suggests that some of the acute and self‐limited types of pain experienced by children today may be associated with factors such as diet, alcohol,^4^ sedentarism,^162^ obesity,^163^ and screen time.^164^ Socioeconomic factors such as maternal education level may also influence the occurrence of pain, with higher pain frequency reported in more disadvantaged children.^165^

Although the primary aim of this analysis was to report the prevalence of acute pain in children, this review also sought to elucidate some of key challenges regarding the assessment of acute pain in children based on the information reported in the prevalence studies. However, the search strategy was not structured to assess the quality of different pain assessment tools used for diagnosing acute pediatric pain. Hence, further investigation should evaluate whether the challenges reported in the prevalence studies are reflective of the real‐world setting and whether these have any implications in clinical practice and research. Most of the studies that reported prevalence of pain types such as headache, abdominal pain, menstrual pain, dental pain, and musculoskeletal pain used pain assessment tools to evaluate acute pain conditions. Clinical examination was used to assess temporomandibular pain in a few studies. Pain assessment tools used were either self‐reported (including faces or numeric rating scales) or parent‐ or physician‐reported (including questionnaires based on ICHD criteria, HBSC symptom checklist, Dental Discomfort Questionnaire, Child Dental Pain Questionnaire, or child or parent interviews conducted by the physician). Self‐reported questionnaires were used in most studies, and parent/carer assessment was used in younger children. Physician assessments were also used for temporomandibular pain. There was heterogeneity in the pain assessment tools utilized. The dearth of prevalence data in children aged under 5 years may be due to a shortage of developmentally appropriate, validated assessment tools—if there is no reliable way to assess pain, the prevalence cannot be reported. The questionnaires did not consistently try to elucidate the risk factors or biological cause of the acute pain; if the underlying issue is not determined, the treating physician may not be able to decide on the best treatment approach—whether it should be pharmacological, nonpharmacological or both. Through the development of more specialized questionnaires that incorporate both physiological and behavioral indicators for different pain types, it may be clearer to select and implement the most appropriate pain‐relieving intervention.

There were very few prevalence studies that highlighted how acute pain in children was treated, what pharmacological and nonpharmacological interventions were available, and why the chosen intervention was selected. The limited data collected here are not unexpected, however, as the search strategy was structured to find studies relating to acute pediatric pain prevalence rather than pain‐relieving interventions. The rate of medical consultation was found to be low. As this review was limited to prevalence studies, there was no mention of guidelines for the diagnosis and treatment of acute pain—challenges regarding the optimal management of acute pain in real‐world clinical practice settings may relate to a lack of standardized guidelines to support healthcare professionals in the effective diagnosis and treatment of acute pain in children. Only a few studies in this analysis reported analgesic use for the treatment of acute pediatric pain. Of these, only three specified what type of painkiller was used—acetaminophen (three studies), aspirin (two studies), and nonsteroidal anti‐inflammatory drugs (three studies).^27^, ^48^, ^53^ Additionally, in some studies, there was a high rate of self‐medication,^16^, ^20^, ^22^, ^27^, ^112^ and this may be likely to increase with age.^27^ Appropriate self‐medication for pain relief must be balanced against the potential risks that come with inappropriate self‐medication, such as adverse reactions or the potential for dependence or abuse.^166^ Females self‐medicated more frequently than males. Menstrual pain appeared to be the only pain type mentioned where nonpharmacological interventions were used to any significant level for pain relief. There may be a lack of pediatric formulations of some analgesics in some countries, and access to medication or health care may be an issue for children of a lower socioeconomic group; this may suggest a negative cycle where problems with health care access led to pain, and in turn, pain is not well managed because of poor access to health care. An increased knowledge of the unmet clinical and medical needs for acute pain management in children, with more insight per age group, would help to identify any gaps in the availability of specific interventions.

As well as there being a need for appropriate formulations and doses of child‐friendly analgesics in the OTC space, there is also a requirement for the education of children and parents/carers on dosage and frequency, when to start and stop treatment, when to consult a healthcare professional, and suitable nonpharmacological alternatives. Although it may be habitual for healthcare professionals, and indeed parents and carers, to only focus on the biological aspect of pain and the ensuing medical treatment, a multimodal management approach could help to ensure that acute pain does not progress to a chronic state. The biopsychosocial model of pain management may be applicable for many pain types, as we identified in our analysis, to counteract the interplay between the biological, psychological, and environmental factors that could influence a child's pain.^167^ If assessment tools consistently included psychological factors, such as symptoms of depression and anxiety, sleep disturbances, and impact on quality of life, this might give a more holistic perspective on the pain that the child is enduring and support with the development of the most appropriate management strategy for that individual. Lifestyle and behavioral changes in addition to pharmacological treatment could help to reduce pain and improve health, regardless of the type of pain. There are also physical techniques, such as meditation and massage, which have been found to relieve pain symptoms and enhance pharmacological strategies.^168^ Educational initiatives and lifestyle guidance should also extend to schools (teachers and school nurses), to ensure that children can identify pain and seek timely support, learn how to self‐medicate, and appreciate the importance of behavioral modifications, such as adopting a better posture when sitting/standing, minimizing school bag weight where possible, and increasing physical exercise.

If a child's pain is not identified, their future pain experiences in terms of their behavioral, sensory, and affective responses may be negatively impacted.^169^ Long‐term consequences of poorly treated pain include a negative impact on quality of life and school performance, including absence when pain is uncontrolled. Children may experience a lowering of their pain threshold and increased pain sensitivity, and analgesics may become less effective.^170^ Cultural factors can influence health care and pain management, and health‐seeking behaviors may be learned or influenced by a cultural or social group, or by family involvement.^171^ By reinforcing to children that their pain matters and will not be ignored, and reversing the stigma that pain should be endured,^1^ they may be more likely to seek support when they are suffering.

Most acute pain research has been conducted on older children or adolescents. This has left gaps in our knowledge about acute pain in the youngest age group, in whom important developmental milestones may not yet have been reached. Surprisingly, little research has been carried out to understand clinically meaningful outcomes and the effectiveness of treatment strategies in younger children.^1^ In addition, none of the prevalence studies we reviewed included children with intellectual or developmental disabilities. Further research is required to understand prevalence rates in an all‐inclusive general population. Most types of pain in children can be described using the same classification system as adults; those that are based on pain duration (i.e., acute versus chronic pain) and underlying pathophysiology (i.e., nociceptive versus neuropathic pain) are used most often.^172^ This raises the question of whether a pediatric classification system would be more appropriate for acute pain, to reflect its multimodal nature and with appropriate terminology and clarity around potential causes, risk factors, and mechanisms. Acute and chronic pain in children should be reviewed from a developmental perspective,^1^ and as such, separate classifications per age groups may even be worth considering, for example, 0–5, 6–11, and 12–18 years. There are new techniques in development that promise to quantify the pain experience in the very young in whom communication of their pain experience is not possible or limited, for example, functional magnetic resonance imaging.^173^ Given that pain is a subjective experience, it will be important that consideration is given to accessibility, timing of treatment, and overall pain perception when looking to validate these tools, in terms of potential clinical utility as tools to determine a more accurate assessment of pediatric pain.

Although this review looked at acute pain prevalence in children by analyzing a large number of studies, there were several limitations that we encountered. The scope of this review was broad, and the studies included were heterogeneous in terms of pain location, age ranges, socioeconomic groups, geography, and methodology. Comparability between studies was complicated by the reporting of different recall periods for when the pain occurred (e.g., point prevalence, weekly or monthly). Pain prevalence studies involving small subsets may not be truly reflective of the general population, hence why the prevalence estimates reported here have such wide ranges. The lack of standardization and robustness of the questionnaires utilized also limited the ability to draw firm conclusions regarding acute pain prevalence. In studies with a pediatric population covering a wide age range, the use of both self‐reported and parent/carer‐assessed pain may have introduced inconsistency and bias; evidence suggests that although parent reports have value, parents may be largely unaware of their child's pain and report their experience inaccurately.^174^, ^175^ There may also be an element of reporting bias if children choose not to communicate their pain because they do not want to bother their parents/carers or are scared at the prospect of visiting a healthcare professional.^168^ Therefore, in some studies in this analysis, owing to the subjective evaluation of pain in very young children by carers, parents, or HCPs, the prevalence estimation of pain in infants may not be completely accurate, but this is unlikely to affect the overall reliability of the results of this review based on the low number of studies identified. Additionally, the effect of specific painkillers or interventions could not be determined owing to a lack of studies that reported how the pain was managed or treated. Finally, very few systematic reviews or meta‐analyses were used in this analysis; therefore, data cannot be interpreted qualitatively; further investment in such articles may be warranted.

The prevalence of acute pain in children and adolescents is high; certain pain types become more prevalent with increasing age, and females tend to experience pain more regularly than males. This review identified several psychosocial correlates of acute pain in children and adolescents, such as lower socioeconomic status and anxiety. Lifestyle habits and behaviors may influence the occurrence of certain types of pain. Health education programs for children, parents/carers, and schools concerning identification and management of acute pain throughout childhood should be a priority, including raising awareness of the potential for under‐recognition of pain in the very young and the potential consequences and implications of under‐treatment.

The paucity of reported information in this area appears to be out of proportion with the prevalence and burden of acute pain in children, and this evidence gap could indicate that clinicians responsible for treating children with acute pain, or other healthcare professionals recommending treatment options, are not yet equipped with an optimal pain management strategy to guide their practice. Untreated pain in childhood can have significant consequences in adulthood; thus, it is essential to introduce preventative measures to reduce the risk of long‐term complications. A more proactive attitude to investigation of child‐reported pain is warranted; timely access to appropriate interventions may aid a faster recovery and reduce the risk of longer‐term complications. Personalizing the management strategy by way of a biopsychosocial approach will ensure that the child is treated holistically according to their unique pain status.

There are challenges associated with obtaining pediatric prevalence data, and applicability of findings from small studies to general populations can be problematic. Further comprehensive research is needed to understand whether the challenges faced at the preliminary level of prevalence studies are the same as those in interventional studies or observational studies, and how this translates to the real‐world setting.

## AUTHOR CONTRIBUTIONS

All authors were involved in the conception of the review, and critically reviewed and commented on the manuscript at all stages of development. All authors approved the final manuscript.

## Supporting information


Appendix S1
