# Supplementary material for: Narrative review of the prevalence and distribution of acute pain in children in the self‐care setting
Source: Paediatr Neonatal Pain. 2022 Aug 8;4(4):169–91. doi: 10.1002/pne2.12085 (PMC9798044; doi:10.1002/pne2.12085)
Supplement: Supplementary file 1 — Appendix S1 [file PNE2-4-169-s001.docx]

## Supplementary materials

A literature search was performed on 28 August 2020 using EmBase. The search terms can be found below. This search produced 776 hits.

**Embase search terms**

('pain'/mj OR 'acute pain':ti,ab OR 'deep pain':ti,ab OR 'lightning pain':ti,ab OR 'nocturnal pain':ti,ab OR 'pain':ti,ab OR 'pain response':ti,ab OR 'pain syndrome':ti,ab OR 'treatment related pain':ti,ab) AND ('pediatrics'/mj OR 'community paediatrics':ti,ab OR 'community pediatrics':ti,ab OR 'paediatric aspect':ti,ab OR 'paediatric care':ti,ab OR 'paediatric educating':ti,ab OR 'paediatric education':ti,ab OR 'paediatric institute':ti,ab OR 'paediatric internship':ti,ab OR 'paediatric perspective':ti,ab OR 'paediatric practice':ti,ab OR 'paediatric research':ti,ab OR 'paediatric service':ti,ab OR 'paediatrics':ti,ab OR 'paediatrics department':ti,ab OR 'pediatric aspect':ti,ab OR 'pediatric care':ti,ab OR 'pediatric educating':ti,ab OR 'pediatric education':ti,ab OR 'pediatric institute':ti,ab OR 'pediatric internship':ti,ab OR 'pediatric practice':ti,ab OR 'pediatric research':ti,ab OR 'pediatric service':ti,ab OR 'pediatrics':ti,ab OR 'pediatrics department':ti,ab OR 'pediatry':ti,ab OR 'social pediatry':ti,ab OR 'well baby clinic':ti,ab OR 'baby'/mj OR 'baby':ti,ab OR 'newborn'/mj OR 'child, newborn':ti,ab OR 'full term infant':ti,ab OR 'human neonate':ti,ab OR 'human newborn':ti,ab OR 'infant, newborn':ti,ab OR 'neonate':ti,ab OR 'neonatus':ti,ab OR 'newborn':ti,ab OR 'newborn baby':ti,ab OR 'newborn child':ti,ab OR 'newborn infant':ti,ab OR 'newly born baby':ti,ab OR 'newly born child':ti,ab OR 'newly born infant':ti,ab OR 'child'/mj OR 'child':ti,ab OR 'children':ti,ab OR 'adolescent'/mj OR 'adolescent':ti,ab OR 'teenager':ti,ab OR 'infant'/mj OR 'infant':ti,ab) NOT ('pregnancy'/exp OR 'child bearing' OR 'childbearing' OR 'gestation' OR 'gravidity' OR 'intrauterine pregnancy' OR 'labor presentation' OR 'labour presentation' OR 'pregnancy' OR 'pregnancy maintenance' OR 'pregnancy trimesters' OR 'pregnant woman'/exp OR 'pregnant woman' OR 'pregnant women' OR 'childbirth'/exp OR 'child birth' OR 'childbirth' OR 'partus' OR 'chronic pain'/exp OR 'chronic intractable pain' OR 'chronic pain' OR 'pain, chronic' OR 'cancer pain'/exp OR 'cancer pain' OR 'malignant pain' OR 'hospital'/exp OR 'clinic' OR 'emergency hospital' OR 'environment, hospital' OR 'hospital' OR 'hospital data' OR 'hospital environment' OR 'hospital establishment' OR 'hospitals' OR 'hospitals, chronic disease' OR 'hospitals, convalescent' OR 'hospitals, group practice' OR 'hospitals, maternity' OR 'hospitals, osteopathic' OR 'hospitals, packaged' OR 'hospitals, paediatric' OR 'hospitals, pediatric' OR 'hospitals, proprietary' OR 'hospitals, satellite' OR 'hospitals, special' OR 'hospitals, state' OR 'infirmary' OR 'medical clinic' OR 'regional hospital' OR 'state hospital' OR 'voluntary hospital' OR 'hospital patient'/exp OR 'hospital patient' OR 'hospitalised patient' OR 'hospitalised patients' OR 'hospitalized patient' OR 'hospitalized patients' OR 'in-hospital patient' OR 'in-hospital patients' OR 'in-patient' OR 'in-patients' OR 'inpatient' OR 'inpatients' OR 'patient, hospital' OR 'hospital care'/exp OR 'hospital care' OR 'intramural care' OR 'medical care, hospital' OR 'palliative therapy'/exp OR 'palliation' OR 'palliative care' OR 'palliative consultation' OR 'palliative medicine' OR 'palliative radiotherapy' OR 'palliative surgery' OR 'palliative therapy' OR 'palliative treatment' OR 'symptomatic treatment' OR 'case report'/exp OR 'case report') AND ('prevalence'/mj OR 'prevalence':ti,ab OR 'prevalence study':ti,ab OR 'age distribution'/mj OR 'age distribution':ti,ab OR 'age structure':ti,ab OR 'distribution, age':ti,ab OR 'epidemiology'/mj OR 'clinical epidemiology':ti,ab OR 'cohort effect':ti,ab OR 'confounding factors (epidemiology)':ti,ab OR 'confounding factors, epidemiologic':ti,ab OR 'controlled before after studies':ti,ab OR 'controlled before and after studies':ti,ab OR 'controlled before and after study':ti,ab OR 'controlled before-after studies':ti,ab OR 'effect modifier, epidemiologic':ti,ab OR 'effect modifiers (epidemiology)':ti,ab OR 'effect modifiers (psychology)':ti,ab OR 'environmental epidemiology':ti,ab OR 'epidemiologic factors':ti,ab OR 'epidemiologic methods':ti,ab OR 'epidemiologic research':ti,ab OR 'epidemiologic research design':ti,ab OR 'epidemiologic studies':ti,ab OR 'epidemiologic study characteristics':ti,ab OR 'epidemiologic study characteristics as topic':ti,ab OR 'epidemiologic survey':ti,ab OR 'epidemiological research':ti,ab OR 'epidemiology':ti,ab OR 'epidemiology model':ti,ab OR 'epidemiometry':ti,ab OR 'healthy worker effect':ti,ab OR 'historically controlled study':ti,ab OR 'interrupted time series analysis':ti,ab OR 'precipitating factors':ti,ab OR 'sampling studies':ti,ab) AND [english]/lim AND [humans]/lim AND [2010-2020]/py AND [embase]/lim.
